# Red and fallow deer determine the density of *Ixodes ricinus* nymphs containing *Anaplasma phagocytophilum*

**DOI:** 10.1186/s13071-020-04567-4

**Published:** 2021-01-19

**Authors:** Katsuhisa Takumi, Tim R. Hofmeester, Hein Sprong

**Affiliations:** 1 Centre for Zoonoses and Environmental Microbiology Centre for Infectious Disease Control, National Institute for Public Health and the Environment (RIVM), Bilthoven, The Netherlands; 2 Department of Wildlife Fish and Environmental Studies, Swedish University of Agricultural Sciences, Skogsmarksgränd 7, 907 36 Umeå, Sweden

## Abstract

**Background:**

The density of *Ixodes ricinus* nymphs infected with *Anaplasma phagocytophilum* is one of the parameters that determines the risk for humans and domesticated animals to contract anaplasmosis. For this, *I. ricinus* larvae need to take a bloodmeal from free-ranging ungulates, which are competent hosts for *A. phagocytophilum.*

**Methods:**

Here, we compared the contribution of four free-ranging ungulate species, red deer (*Cervus elaphus*), fallow deer (*Dama dama*), roe deer (*Capreolus capreolus*), and wild boar (*Sus scrofa*), to *A. phagocytophilum* infections in nymphs. We used a combination of camera and live trapping to quantify the relative availability of vertebrate hosts to questing ticks in 19 Dutch forest sites. Additionally, we collected questing *I. ricinus* nymphs and tested these for the presence of *A. phagocytophilum.* Furthermore, we explored two potential mechanisms that could explain differences between species: (i) differences in larval burden, which we based on data from published studies, and (ii) differences in associations with other, non-competent hosts.

**Results:**

Principal component analysis indicated that the density of *A. phagocytophilum*-infected nymphs (DIN) was higher in forest sites with high availability of red and fallow deer, and to a lesser degree roe deer. Initial results suggest that these differences are not a result of differences in larval burden, but rather differences in associations with other species or other ecological factors.

**Conclusions:**

These results indicate that the risk for contracting anaplasmosis in The Netherlands is likely highest in the few areas where red and fallow deer are present. Future studies are needed to explore the mechanisms behind this association.

**Graphical abstract:**

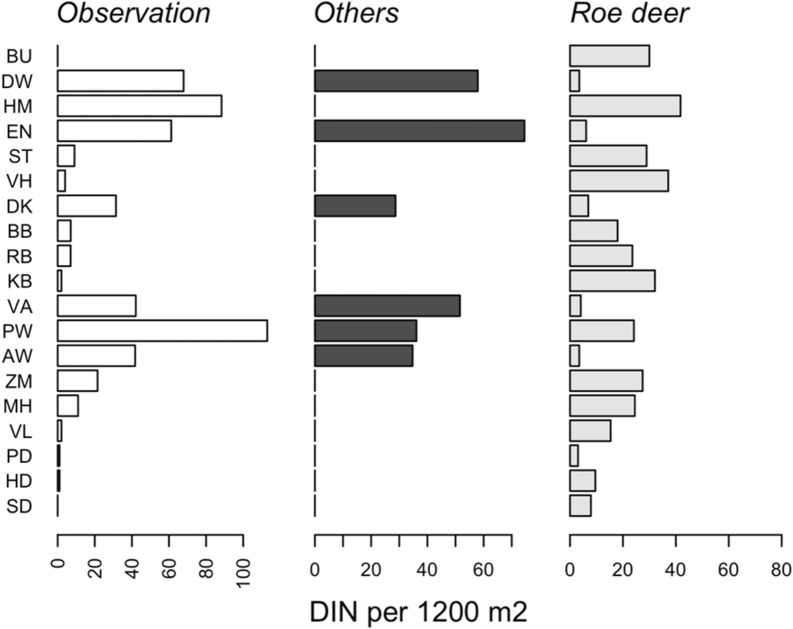

## Background

*Anaplasma phagocytophilum* is the causative agent of human granulocytic anaplasmosis (HGA), and it also causes disease and economic losses in domesticated animals [1–3]. The first human case in Europe was reported in 1995. Since then, HGA cases have only been occasionally reported throughout Europe [4]. It is unclear to what extent HGA poses a health burden in Europe: epidemiological data on the disease incidence and disease burden are either incomplete or lacking from most European countries [2]. The non-specificity of the reported symptoms, poor diagnostic tools, and lack of awareness of public health professionals may also complicate these estimations [5]. *Anaplasma phagocytophilum* is transmitted by the bite of an infected tick [6, 7]. The main vector in Europe is *Ixodes ricinus,* which also transmits *Borrelia burgdorferi* (*s.l.*), the causative agent of Lyme borreliosis, and several other pathogens [8]. The geographic spread and density of *I. ricinus* infected with *A. phagocytophilum* are important determinants of the disease risk [9, 10]. *Anaplasma phagocytophilum* seems to appear in all countries across Europe with infection prevalence in nymphs (NIP) varying between and within countries from 0 to 25% [1]. Understanding which factors determine the spatial and temporal distribution of *I. ricinus* infected with *A. phagocytophilum* is needed for risk assessments and for formulating possible intervention strategies.

Variations in the density of questing *I. ricinus* infected with *A. phagocytophilum* (DIN) have partly been attributed to environmental factors such as differences in weather conditions [11, 12], habitat characteristics [13], as well as vertebrate communities [14]. Whereas small mammals and birds are considered to feed the majority of immature *I. ricinus,* ungulates act as their main propagation host [15]. It is, however, still unclear which host species form the main reservoir for *A. phagocytophilum* and therefore contribute most to the density of infected ticks. *Anaplasma phagocytophilum* has been found to infect many vertebrate species [1], but its genetic diversity indicates that there are multiple genetic variants, or ecotypes, with distinct but overlapping transmission cycles, pathogenicity, and geographical origin [16–18]. A variety of wildlife species, like red deer (*Cervus elaphus*), fallow deer (*Dama dama*), wild boar (*Sus scrofa*), and European hedgehog (*Erinaceus europaeus*), are harbouring *A. phagocytophilum* variants that can cause disease in humans and domesticated animals, whereas roe deer (*Capreolus capreolus*), rodents, and birds seem to carry genetic variants that have until now not been associated with human disease [17].

About two-thirds of tick bites reported in The Netherlands are *I. ricinus* nymphs [19]. Therefore, the density of questing nymphs infected with *A. phagocytophilum* (DIN) is an important ecological parameter that, together with the level of human exposure, determines tick-borne disease risk [9, 20]. The DIN is calculated by multiplying the density of questing *I. ricinus* nymphs (DON) by nymphal infection prevalence (NIP). The transmission of *A. phagocytophilum* predominantly relies on horizontal transmission between ticks and vertebrate hosts and on transstadial transmission in its vectors, as vertical (transovarial) transmission has not been documented for *I. ricinus.* Therefore, an *I. ricinus* larva needs to take a bloodmeal from an infected vertebrate host to become an infected *I. ricinus* nymph. The availability of (infected) vertebrates to questing larvae generally drives the density of (infected) *I. ricinus* nymphs [14, 21, 22].

Using data from a cross-sectional study estimating the availability of hosts with camera and live traps in 19 Dutch forest sites, we quantified a moment when a questing tick encounters an ungulate; the probability of this event predicts both *I. ricinus* nymphal density and *A. phagocytophilum* DIN [14]. However, the reported association for *A. phagocytophilum* DIN left a relatively large proportion of the variation among sites unexplained, which could be because of the grouping of four ungulate species. The group of ungulates considered consisted of four species that differed in their ecology and potentially in their ability to be hosts for *I. ricinus* and *A. phagocytophilum.*

Here, we present a re-analysis of the *A. phagocytophilum* data from the cross-sectional study to disentangle the role of the four ungulate species in determining *A. phagocytophilum* DIN. We first applied a principal component analysis to the camera and live trapping data to test if availability of any of the four ungulate species or combinations of species was associated with *A. phagocytophilum* DIN. Second, we used simple mathematical models to explore two potential mechanisms that could explain differences between species: (i) differences in *I. ricinus* larval burden and (ii) potential associations of the different ungulate species with alternative incompetent host species.

## Methods

### Cross-sectional study

We made use of data from an extensive field survey that was carried out in 19 1-ha sites located in forested areas in The Netherlands in 2013 and 2014. Data were collected on the density of questing *I. ricinus* (blanket dragging), vertebrate communities (camera and live trapping), and infection rates of tick-borne pathogens (qPCR detection). The sites, methodologies, and data have been described elsewhere as well as a series of detailed analyses [14, 22–24].

### Host attribution

We arranged the encounter probabilities for all forest sites (*n* = 19) and vertebrate species (*n* = 32) into a matrix $\mathbf{A}\in {\mathbb{R}}^{19\times 32}$ having 19 rows and 32 columns. We further arranged *A. phagocytophilum* DIN into a vector $b$ matching the order of the forest sites along the rows of $\mathbf{A}$. To attribute *A. phagocytophilum* DIN to an assemblage of 32 vertebrate species, we factored the matrix $\mathbf{A}$ into two orthogonal matrices

$$\mathbf{U}=[{u}_{1},{u}_{2},\dots ,{u}_{19}]\in {\mathbb{R}}^{19\times 19} \mathrm{and} \mathbf{V}=[{v}_{1},{v}_{2},\dots ,{v}_{32}]\in {\mathbb{R}}^{32\times 32}$$

and a diagonal matrix ${\varvec{\Sigma}}\in {\mathbb{R}}^{19\times 32}$ with the singular values ${\sigma }_{1}\ge {\sigma }_{2}\dots \ge {\sigma }_{19}\ge 0$ and the remaining entries equal to zero. Theorem 2.5.2 [25] proves that $\mathbf{A}=\mathbf{U}{\varvec{\Sigma}}{\mathbf{V}}^{T}$. The column vectors ${u}_{i}$ and ${v}_{i}$ are principal components, also known as singular vectors.

The *A. phagocytophilum* DIN increased at each forest site because of the first principal components by the amount (Theorem 5.5.1 [25])

1 $$\frac{{u}_{1}\cdot b}{{\sigma }_{1}}\mathbf{A}{v}_{1}\in {\mathbb{R}}^{19}.$$

We attributed Eq. (1) to roe deer because the highest contribution from the first principal component ${v}_{1}$ comes from roe deer. Next, we applied the theorem again to quantify the inputs from the lower principal components ${v}_{2},{v}_{3}\dots$,

$$y=\sum_{i=2}^{8}\frac{{u}_{i}\cdot b}{{\sigma }_{i}}\mathbf{A}{v}_{i}\in {\mathbb{R}}^{19}.$$

Lower components ${v}_{9}\dots {v}_{19}$ are ignored because the tail sum $\sum_{i=9}^{19}{\sigma }_{i}^{2}$ is negligible (2.43%) compared to the whole cumulative sum $\sum_{i=2}^{19}{\sigma }_{i}^{2}$. Next, we define

2 $$\begin{array}{cc}{y}^{+}& =\mathrm{max}(y,0),\\ & \end{array}$$

3 $$\begin{array}{cc}& \\ {y}^{-}& =\mathrm{max}(-y,0).\end{array}$$

Intuitively, we clipped the solution $y$ into the positive part ${y}^{+}$ and the negative part ${y}^{-}$. We attributed Eq. (2) to fallow deer, red deer, and wild boar because the highest contributions from the second principal component ${v}_{2}$ and the third ${v}_{3}$ come from these species.

### Larval tick burden on ungulates

Ungulates generally contribute relatively little as hosts for feeding *I. ricinus* larvae compared to rodents and birds [15]. Nevertheless, as important hosts for *A. phagocytophilum,* ungulates might feed a significant fraction of larvae that later become *A. phagocytophilum*-infected nymphs. Thus, differences in larval burden between ungulate species could contribute to differences in their importance as hosts contributing to *A. phagocytophilum* DIN. To explore this, we compiled data from published studies that collected *I. ricinus* larvae attached to individual ungulates (see Additional file 1: Table S1). We extracted species, the number of checked animals, and the number of *I. ricinus* larvae attached to the animals. We fit the negative binomial model (log-link) to the number of larval ticks using the number of animals and the species as predicting variables. We tested the significance of the species predicting variable by performing the likelihood ratio test.

### Associations with other woodland species

We explored whether differences between species could be explained by associations of the different ungulate species with the availability of other host species. Here, two species might appear related because of the probability condition (the sum of rates over the host species must equal to one). To remove this potential bias, we performed the following analysis using the encounter rates instead of the encounter probability. For this, we calculated the Pearson correlation in the encounter rate for each ungulate species with each other woodland species. We fit the binomial model (logit link) to the frequency of positive and negative correlation values using the ungulate species as a predicting variable. We tested the significance of the predicting variable by performing the likelihood ratio test.

Absent species interaction, correlation values should be close to zero, and the deviation from the expected value zero should be symmetric. It is possible to calculate the probability of observing as many or more negative correlation values as actually observed in the vertebrate community,

4 $$\frac{1}{{2}^{-n}}\sum_{j=k}^{n}\left(\genfrac{}{}{0pt}{}{n}{j}\right).$$

This is a partial sum of binomial probability densities where a correlation value is negative with probability $\frac{1}{2}$. The vertebrate community counts $n$ members. The number of negative correlation values observed in the vertebrate community equals $k$. All computations were implemented using the R language [26].

## Results

### Tick densities

We collected a total of 16,568 *I. ricinus* nymphs at the 19 forest sites. Most of the collected nymphs (*n* = 13,967) were tested for the presence of *A. phagocytophilum* DNA resulting in an overall infection prevalence of 3.3 % (456 infected nymphs), which we used to calculate the density of infected nymphal ticks (DIN). Using correlation analyses, we detected a significant correlation between the number of positive nymphs, NIP, and DIN (Table 1). DIN of *A. phagocytophilum* lacked a clear correlation with the density of nymphal ticks (DON; Table 1).

**Table 1:** Density of nymphs infected with A. phagocytophilum (DIN) is unrelated with the density of nymphs (DON)

	DON	Test	Positive	NIP	DIN
DON					
Test	*0.7*				
Positive	0.26	*0.48*			
NIP	0.11	0.22	*0.93*		
DIN	0.34	0.41	*0.94*	*0.91*	

Table entries are Pearson correlations calculated using Additional file 1: Table S2. Numerals are italicized when the *p*-value is < 0.05. DON: Density of questing *Ixodes ricinus* nymphs. Test: Number of questing *Ixodes ricinus* nymphs tested for the presence of *Anaplasma phagocytophilum.* Positive: Number of *Anaplasma phagocytophilum* presence in questing *Ixodes ricinus* nymphs

*NIP* Positive divided by test, *DIN* density of infected *Ixodes ricinus* nymphs.

### Host attribution

**Fig. 1:**
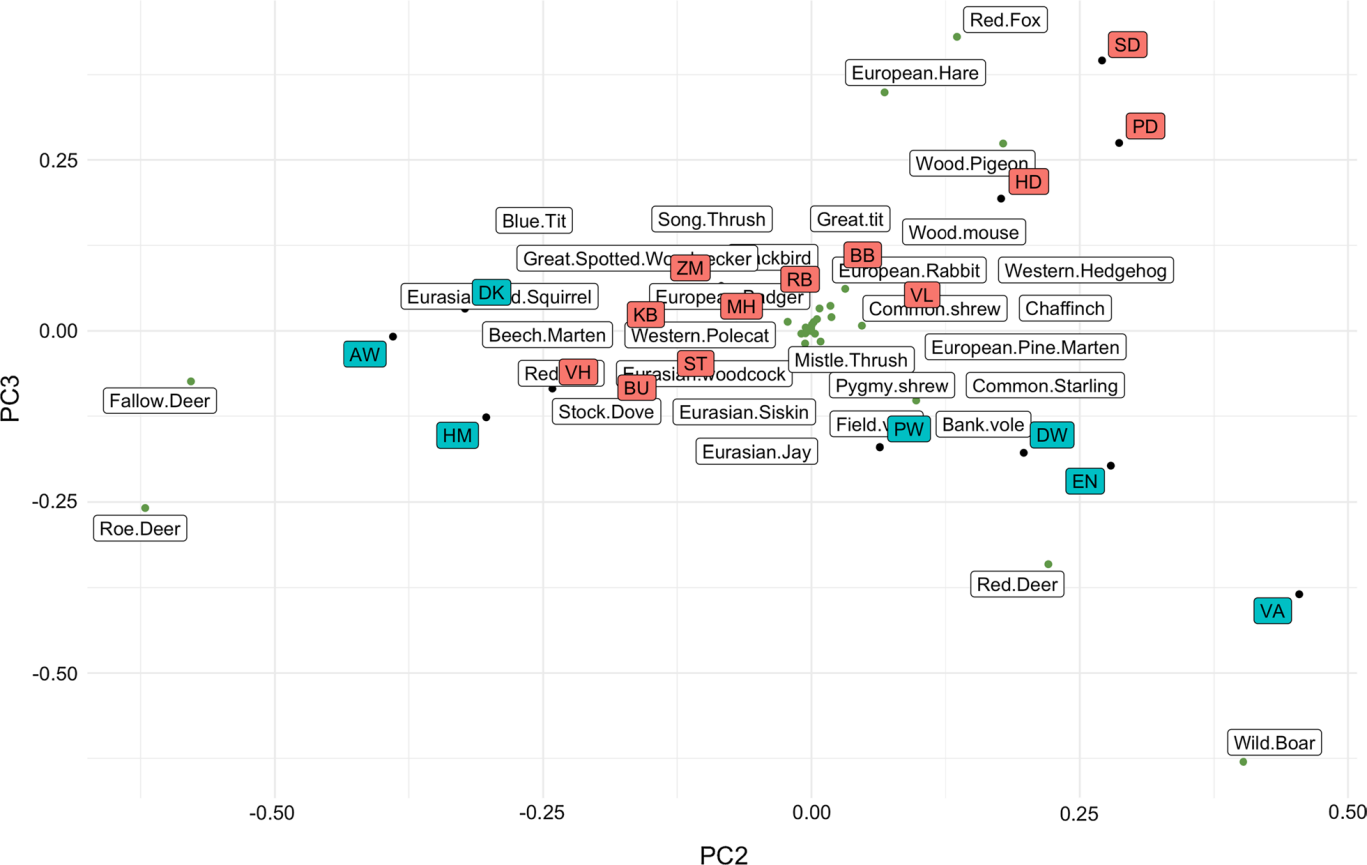
PCA plot of encounter probabilities. A black dot is a position of a forest site in 2nd and 3rd PCA coordinates. A box contains two letters abbreviating a forest site name. A green box indicates a high *Anaplasma phagocytophilum* DIN. A red box dicates a low *Anaplasma phagocytophilum* DIN. A green dot is a position of a woodland species. The species name is placed in a distance away from the green dot to avoid excessive overlaps

**Fig. 2:**
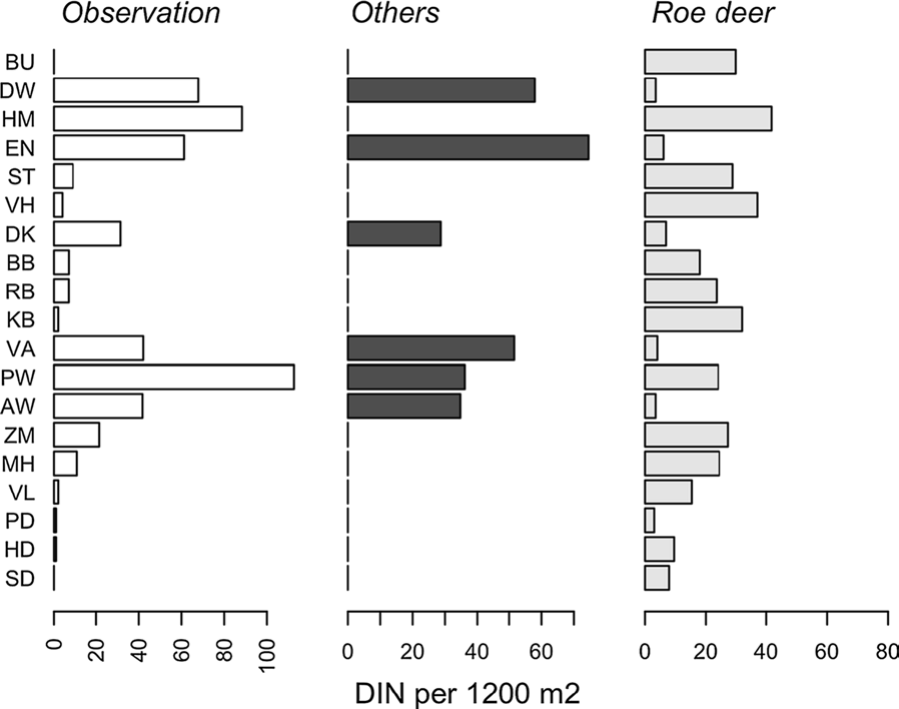
The three free-ranging ungulates except roe deer support *Anaplasma phagocytophilum* DIN. Observed *Anaplasma phagocytophilum* DIN was calculated by multiplying the density of questing *Ixodes ricinus* nymphs by the *Anaplasma phagocytophilum* NIP. Bar heights in the middle panel (others referring to fallow deer, red deer, and wild boar) were calculated using Eq. (2). Bar heights in the right panel were calculated using Eq. (1)

The encounter of an *I. ricinus* larva with a woodland species is a critical event to a successful *A. phagocytophilum* transmission. We quantified the probability of an encounter event based on the information collected using camera- and live-traps in the 19 forest sites (see Additional file 1: Fig S1). We found that the majority of the observed *A. phagocytophilum* DIN can be attributed to the encounter probabilities of fallow deer, red deer, and wild boar (Fig. 1), based on the correlation of the attributed DIN to the three free-ranging ungulate species (Fig. 2). We, however, did not find a correlation with the attributed DIN to roe deer (Table 2).

**Table 2:** Observed *Anaplasma phagocytophilum* DIN lacks a correlation with roe deer and correlates with the other fee-ranging ungulates

	Observation	Others	Roe deer
Observation			
Others	*0.64*		
Roe deer	− 0.02	− *0.56*	

Table entries are Pearson correlations. Numerals are italicized when the *p*-value is < 0.05

### Larval tick burden on ungulate species

Based on the data extracted from 12 studies in the literature  [27–38], which together report 24,794 *I. ricinus* larvae attached to 1860 individual ungulates in 6 European countries, we found no support for the hypothesis that differences in *I. ricinus* larval burden could have caused the differences in association with *A. phagocytophilum* DIN among the four ungulate species (Table 3).

**Table 3:** Analysis of larval ticks on four ungulate species

Model	Theta	Resid. df	2 x log-lik.	Test	df	LR stat.	Pr(chi)
Samples	0.289	18	− 293.764				
Samples + Species	0.317	15	− 291.179	1 *vs* 2	3	2.585	0.46

Additional file 1: Table S1 displays the data.

### Reducing availability of woodland species

The encounter rate of roe deer correlated negatively with the encounter rates of 14 woodland species and positively with 17 (Table 4). The probability of exceeding the number of negative correlations is 0.81. The other free-ranging ungulates showed a different pattern: Encounter rates of red deer correlated negatively with encounter rates of 23 woodland species, fallow deer with 26 woodland species, and wild boar with 23 woodland species (Table 4). Consequently, we found a clear difference among these ungulate species in the frequency of negative correlations with other woodland species (*p*-value = 0.010623, deviance = 11.2140483, d.f. = 3). The probability of exceeding the number of negative correlation is low: red deer: 0.01; fallow deer: 0.00027: wild boar: 0.01.

**Table 4:** Frequency of positive and negative correlations in encounter rates with 32 woodland species

	Positive	Negative
Roe deer	18	14
Fallow deer	6	26
Red deer	9	23
Wild boar	9	23

A cell displays a number of woodland species having a positive (or negative) correlation with the ungulate species. Additional file 1: Table S3 displays the data.

## Discussion

**Fig. 3:**
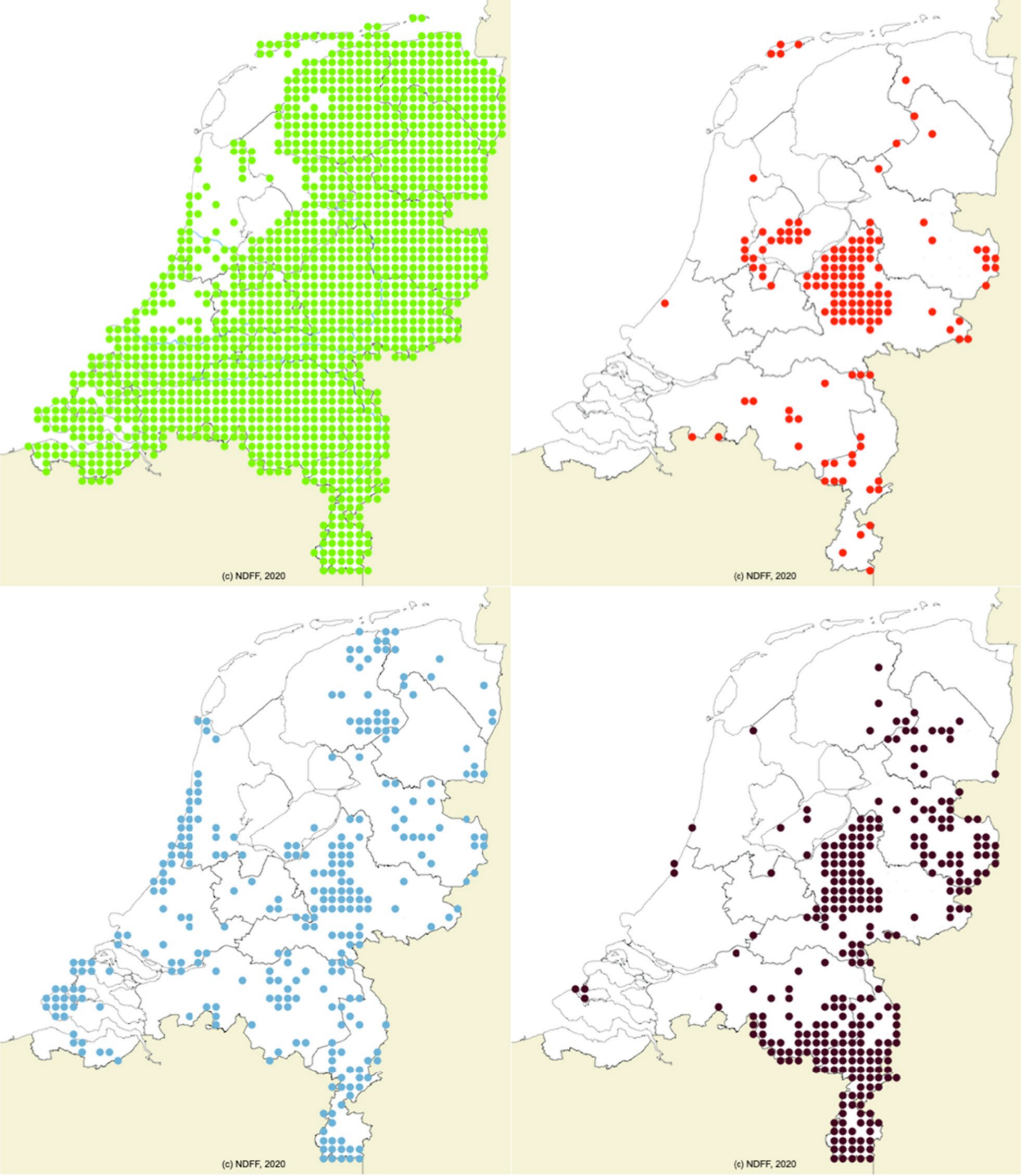
Ungulate presence: The Netherlands 2000–2020 (Source: https://www.verspreidingsatlas.nl Green: roe deer. Red: red deer. Blue: fallow deer. Black: wild boar)

Re-analysing a cross-sectional study of observed *A. phagocytophilum* DIN at 19 Dutch forest sites [14], we show that most of the variation in DIN can be explained by differences in encounter probability among ungulates species that were not taken into account in the original analysis. We found a clear association of *A. phagocytophilum* DIN with the encounter probabilities of fallow deer, red deer, and wild boar. A first exploration of two potential mechanisms that could cause the difference among ungulate species in their contribution to the DIN indicated that negative association of fallow deer, red deer, and wild boar with the encounter rate of other woodland species is a likely candidate for the found differences. In contrast, we did not find support for an explanation based on differences in *I. ricinus* larval burden. In The Netherlands, fallow deer, red deer, and wild boar only occur in a few specific areas (Fig. 3). Therefore, we cannot rule out if found associations with other woodland species are a result of ecological (interspecies) interactions or species associations with different habitats and wildlife management. It is, however, clear that forested areas where fallow deer, red deer, or wild boar occur likely have the highest risk for humans and domesticated animals to be exposed to *A. phagocytophilum* though tick bites. Targeted health campaigns to increase the awareness amongst health professionals might help to identify HGA cases and may further be used for stimulation of preventive measures.

### Previous research linking species difference to Anaplasma phagocytophilum

We did not find differences among ungulates in their *I. ricinus* larval burden based on published studies. However, the number of studies was limited (*n* = 12), especially for some species (*n* = 1 for fallow deer and wild boar). Thus, the lack of differences is likely a result of large variation in larval burdens and limited sample sizes, especially as studies comparing *I. ricinus* burden among different ungulate species in single study sites did find differences among species [27, 28]. This indicates that there is a need for more comparative studies investigating differences in the *I. ricinus* burden of the different ungulates related to differences in infection prevalence with *A. phagocytophilum* in both hosts and feeding ticks.

We found a negative association in the encounter rates of fallow deer, red deer, and wild boar with the encounter rates of other woodland species, which could be caused by both ecological interactions and management practices. Contrary to roe deer, these three species have a very limited distribution in The Netherlands occurring mainly in the dunes along the coast (fallow deer) and on a large forested area in the middle of The Netherlands called the Veluwe (Fig. 3). This restricted distribution is completely due to present and past wildlife management [39]. Both of these areas are characterized by relatively high sandy soils resulting in relatively low productivity [40]. As a result, both the coastal dunes and the Veluwe harbour a limited number of species, which could explain the negative associations we found. Simultaneously, there could be additional interspecific interactions explaining some of our results. An experimental study [41] reported evidence for cascading effects by ungulates on a temperate forest ecosystem. Experimentally excluding fallow deer, roe deer, red deer, wild boar, and mouflon (*Ovis orientalis*) from a temperate forest ecosystem in the Veluwe decreased the density of soil grains and increased litter depth, invertebrate biomass, and rodent activity. Future studies are needed to test for the contribution of either mechanism on the found negative associations.

The bacterium *A. phagocytophilum* is divided into four ecotypes based on the *groEL* sequences and the host association [16]. These ecotypes differ in their zoonotic potential, with ecotype I being able to cause HGA in humans and anaplasmosis in domesticated animals. This is concerning, as red and fallow deer are considered host species for ecotype I [16]. Thus, the peaks in *A. phagocytophilum* DIN in areas with a high encounter probability with fallow deer and red deer indicate that these nymphs are likely infected with ecotype I. In contrast, roe deer are considered hosts for the non-zoonotic ecotype II. As the geographic distribution of fallow and red deer in The Netherlands (Fig. 3) is far more restricted than the distribution of roe deer, this would imply that the risk of acquiring HGA is largely restricted to areas where red/fallow deer are present. This might be one of explanations for the low incidence of (reported) HGA cases.

Strikingly, we did not find a correlation of *A. phagocytophilum* DIN at the forest sites with the encounter probability of a questing nymph with roe deer (Fig. 2). This contrasts with the finding that the density of questing nymphal ticks in the same forest sites correlated with the encounter probability [14]. Thus, we detected in this cross-sectional study some degree of impedance in the *A. phagocytophilum* life cycle and no evidence of any impedance in the tick life cycle at the forest sites where roe deer is the predominant competent species. This appears to be a common situation in the Dutch forest areas with some exceptions. We observed a high *A. phagocytophilum* DIN at the forest site Halfmijl (HM) (Fig. 2). Only roe deer and none of the other free-ranging ungulates were captured by the camera trapping at the forest site.

There are additional aspects of *A. phagocytophilum* transmission, which could influence *A. phagocytophilum* DIN in theory. Questing nymphs may have been infected by *A. phagocytophilum* while co-feeding as an uninfected larva next to infected nymphs on the same deer. Next, not all variants of *A. phagocytophilum* might persist to the same extent during the off-host period. The low DIN associating with roe deer in our dataset might be due to a lower persistence of Ecotype II in *I. ricinus* during the off-host period. With regard to co-infection, *A. phagocytophilum* causes a chronic/systemic infection in deer, implying that the subsequent larvae feeding on deer will become infected [16]. The latter will be substantially more than the few larvae feeding at the same time with an infected nymph. With regard to differential persistence, a study of *A. phagocytophilum* Ecotypes in Central Europe did not find less Ecotype II than Ecotype I in questing *I. ricinus* nymphs originating from a site in the presence of roe deer [13]. For these reasons, co-feeding and differential persistence were omitted from the analyses.

## Conclusion

In conclusion, our re-analysis of the cross-sectional study suggests that the risk of contracting anaplasmosis in the forest with fallow deer, red deer, and wild boar is high compared to the remaining forest sites where roe deer is the predominant competent host. Geographical distribution of deer species in The Netherlands implies that the risk of acquiring HGA is largely restricted to areas where red/fallow deer are present. Additional studies are required to test the inference based on our associative study.

## Supplementary Information

**Additional file 1.** Additional figure and tables.

## Data Availability

The datasets used and/or analysed during the current study are available from the corresponding author on reasonable request

## Abbreviations

HGA: Human granulocytic anaplasmosis
DON: Density of questing *Ixodes ricinus* nymphs
DIN: Density of infected nymphs
NIP: Infection prevalence in nymphs
