# Supplementary material for: Red and fallow deer determine the density of Ixodes ricinus nymphs containing Anaplasma phagocytophilum
Source: Parasit Vectors. 2021 Jan 19;14:59. doi: 10.1186/s13071-020-04567-4 (PMC7814456; doi:10.1186/s13071-020-04567-4)
Supplement: Supplementary file 1 — Additional file 1. Additional figure and tables. [file 13071_2020_4567_MOESM1_ESM.docx]

# Additional file 1


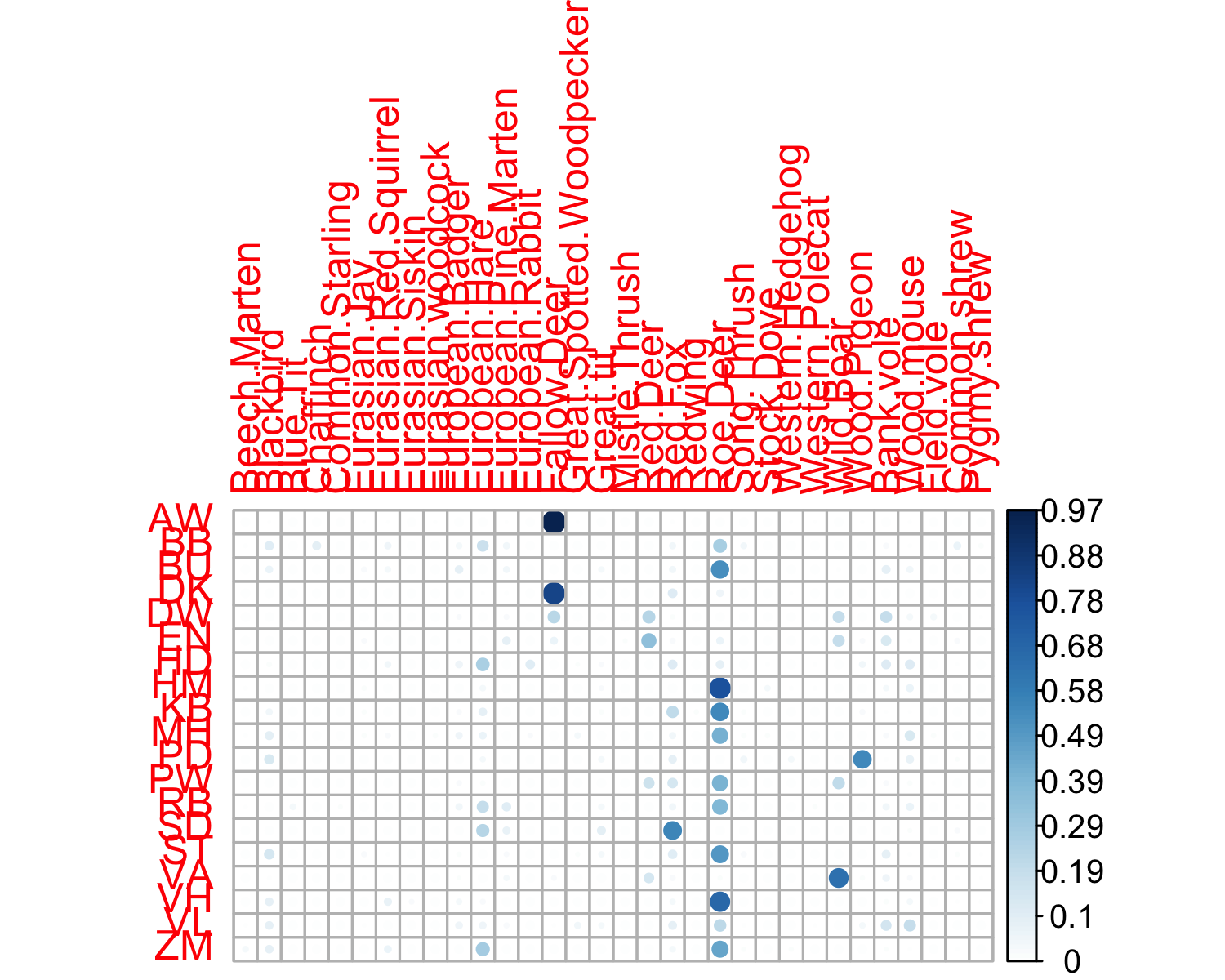


Figure S1: Encounter probability [[14](#ref-pmid31492171)] for each forest site (row) and an individual vertebrate species (column) increases from 0 (light circle) to 1 (dark circle). A row sum equals to 1.

Table S1: Number of larval ticks on four ungulate species.

| Species | Samples | DOL | Reference |
| --- | --- | --- | --- |
| Fallow.deer | 115 | 5719 | [[28](#ref-pmid8517488)] |
| Red.deer | 32 | 0 | [[32](#ref-pmid22260900)] |
| Red.deer | 147 | 4140 | [[28](#ref-pmid8517488)] |
| Red.deer | 38 | 305 | [[27](#ref-pmid25113977)] |
| Red.deer | 49 | 70 | [[33](#ref-pmid26450650)] |
| Red.deer | 33 | 256 | [[34](#ref-pmid25391754)] |
| Red.deer | 197 | 416 | [[35](#ref-pmid23541678)] |
| Wild.boar | 9 | 73 | [[27](#ref-pmid25113977)] |
| Roe.deer | 35 | 3 | [[32](#ref-pmid22260900)] |
| Roe.deer | 224 | 621 | [[35](#ref-pmid23541678)] |
| Roe.deer | 80 | 862 | [[36](#ref-pmid20204470)] |
| Roe.deer | 67 | 577 | [[28](#ref-pmid8517488)] |
| Roe.deer | 154 | 459 | [[37](#ref-pmid24063094)] |
| Roe.deer | 37 | 9805 | [[38](#ref-pmid9423270)] |
| Roe.deer | 367 | 1052 | [[39](#ref-pmid20963472)] |
| Roe.deer | 142 | 268 | [[40](#ref-pmid20099011)] |
| Roe.deer | 2 | 130 | [[41](#ref-pmid32211989)] |
| Roe.deer | 50 | 0 | [[41](#ref-pmid32211989)] |
| Roe.deer | 54 | 8 | [[41](#ref-pmid32211989)] |
| Roe.deer | 28 | 30 | [[41](#ref-pmid32211989)] |

Table S2: Summary of tests for the presence of *Anaplasma phagocytophilum* in questing *Ixodes ricinus* nymphs.

|  | DON | Test | Positive | NIP | DIN |
| --- | --- | --- | --- | --- | --- |
| BU | 2200 | 688 | 0 | 0.000 | 0.000 |
| DW | 1647 | 1650 | 68 | 0.041 | 67.876 |
| HM | 1531 | 624 | 36 | 0.058 | 88.327 |
| EN | 1379 | 1397 | 62 | 0.044 | 61.201 |
| ST | 1134 | 1129 | 9 | 0.008 | 9.040 |
| VH | 1039 | 1039 | 4 | 0.004 | 4.000 |
| DK | 870 | 858 | 31 | 0.036 | 31.434 |
| BB | 869 | 742 | 6 | 0.008 | 7.027 |
| RB | 859 | 863 | 7 | 0.008 | 6.968 |
| KB | 794 | 791 | 2 | 0.003 | 2.008 |
| VA | 765 | 763 | 42 | 0.055 | 42.110 |
| PW | 760 | 767 | 114 | 0.149 | 112.960 |
| AW | 726 | 678 | 39 | 0.058 | 41.761 |
| ZM | 680 | 665 | 21 | 0.032 | 21.474 |
| MH | 640 | 639 | 11 | 0.017 | 11.017 |
| VL | 403 | 398 | 2 | 0.005 | 2.025 |
| PD | 130 | 130 | 1 | 0.008 | 1.000 |
| HD | 120 | 120 | 1 | 0.008 | 1.000 |
| SD | 22 | 26 | 0 | 0.000 | 0.000 |

Table S3: Correlation in encounter rates between ungulates and other forest species [[14](#ref-pmid31492171)].

|  | Roe.Deer | Fallow.Deer | Red.Deer | Wild.Boar |
| --- | --- | --- | --- | --- |
| Beech.Marten | 0.345 | -0.130 | -0.215 | -0.150 |
| Blackbird | 0.325 | -0.180 | -0.358 | -0.200 |
| Blue.Tit | 0.266 | -0.068 | -0.111 | -0.078 |
| Chaffinch | -0.071 | -0.068 | -0.111 | -0.078 |
| Common.Starling | 0.266 | -0.068 | -0.111 | -0.078 |
| Eurasian.Jay | 0.185 | 0.100 | 0.357 | 0.265 |
| Eurasian.Red.Squirrel | 0.108 | -0.069 | -0.316 | -0.220 |
| Eurasian.Siskin | 0.244 | -0.068 | -0.111 | -0.078 |
| Eurasian.woodcock | -0.231 | 0.872 | -0.153 | -0.106 |
| European.Badger | 0.247 | -0.228 | -0.133 | -0.051 |
| European.Hare | 0.189 | -0.196 | -0.320 | -0.224 |
| European.Pine.Marten | -0.037 | -0.033 | 0.475 | 0.344 |
| European.Rabbit | -0.145 | -0.075 | -0.123 | -0.086 |
| Fallow.Deer | -0.327 | 1.000 | -0.110 | -0.083 |
| Great.Spotted.Woodpecker | 0.233 | 0.034 | -0.187 | -0.130 |
| Great.tit | 0.358 | -0.220 | -0.363 | -0.253 |
| Mistle.Thrush | -0.207 | -0.059 | 0.661 | 0.085 |
| Red.Deer | -0.435 | -0.110 | 1.000 | 0.685 |
| Red.Fox | 0.024 | -0.004 | -0.247 | -0.090 |
| Redwing | 0.293 | -0.068 | -0.111 | -0.078 |
| Roe.Deer | 1.000 | -0.327 | -0.435 | -0.325 |
| Song.Thrush | 0.085 | -0.189 | -0.312 | -0.217 |
| Stock.Dove | 0.326 | -0.116 | -0.191 | -0.133 |
| Western.Hedgehog | -0.259 | 0.317 | -0.222 | -0.155 |
| Western.Polecat | 0.256 | 0.073 | -0.240 | -0.167 |
| Wild.Boar | -0.325 | -0.083 | 0.685 | 1.000 |
| Wood.Pigeon | -0.299 | -0.047 | -0.087 | -0.039 |
| Bank.vole | -0.065 | -0.198 | 0.459 | 0.253 |
| Wood.mouse | 0.193 | -0.094 | -0.144 | -0.057 |
| Field.vole | -0.306 | -0.070 | 0.458 | 0.248 |
| Common.shrew | -0.191 | -0.169 | 0.331 | 0.164 |
| Pygmy.shrew | -0.069 | -0.121 | 0.067 | 0.209 |
